# Superior Sagittal Sinus Tumor Eroding through the Skull: An Unfamiliar Presentation of Hepatocellular Carcinoma and Literature Review

**DOI:** 10.1155/2019/5945726

**Published:** 2019-11-04

**Authors:** K. W. Sadik, H. Dayoub, H. Bonatti

**Affiliations:** ^1^Department of Surgery, Guthrie Clinic, 1 Guthrie Sq., Sayre, PA 18840, USA; ^2^Meritus Surgical Specialists, Hagerstown, MD 21742, USA

## Abstract

**Background:**

Hepatocellular carcinoma (HCC) most commonly develops in patients with liver cirrhosis caused by hepatitis C and B virus. HCC is the most common cause of death in people with cirrhosis. The lungs, bone, and lymph nodes are frequent sites of metastasis of HCC.

**Patients and Methods:**

We present a case of solitary skull metastasis as the first symptom of HCC. A literature review with regard to HCC skull metastasis was undertaken.

**Results:**

This patient had HCC based on chronic hepatitis C. He presented with an erosive lesion masquerading as a scalp lipoma resulting in hepatocellular carcinoma with retrograde reflux and sinus metastases. The lesion was ultimately resected, but the patient died within one week from liver failure. Seventeen similar cases were found in the literature. Median age of the patients was 58 years with 95% being male and 35% having viral hepatitis. Surgery was the preferred treatment with chemotherapy and radiation being other options. Outcome was acceptable but baseline liver disease is a limiting factor.

**Discussion:**

Cranial metastases of HCC are rare but should be considered in patients with chronic hepatitis C presenting with scalp skin lesions.

## 1. Introduction

Hepatocellular carcinoma (HCC) is the most common type of primary liver cancer in adults and is the most common cause of death in people with cirrhosis [1]. It occurs in the setting of chronic liver inflammation and is most closely linked to chronic viral hepatitis infection (hepatitis B or C) or exposure to toxins such as alcohol or aflatoxin [2]. With the rise of obesity rates worldwide, HCC based on nonalcoholic steatohepatitis (NASH) has become a major concern in public health. As with any cancer, the treatment and prognosis of HCC vary depending on the specifics of tumor histology, size, how far the cancer has spread, and overall health. The vast majority of HCC occurs in Asia and sub-Saharan Africa, in countries where hepatitis B infection is endemic and many are infected from birth. The incidence of HCC in the US and other developing countries is increasing due to an increase in hepatitis C virus infections although new and very effective treatments have become available and NASH as well as immigration of individuals who are chronic carriers of HBV. HCC is more common in males than in females for unknown reasons [2].

Extrahepatic metastatic spread of HCC occurs in about 50% of cases, most often to the lungs, lymph nodes, and bones. Prognosis of patients with distant metastases from HCC is unfavorable with a median survival time after diagnosis of 6.7 months [3]. In recent years, the metastatic behavior of HCC has shown two interesting new features: First is the spread to unusual sites such as soft tissue [4, 5], the maxillary sinus [6], the orbit [7], and the brain [8]. Second is the identification of the metastatic lesion before the primary tumor is diagnosed [9–13].

In many patients with bone metastases, antineoplastic drug therapy, radiotherapy, and palliative therapy are selected to relieve pain, reduce neurological symptoms, and maintain quality of life. Surgery is indicated for a limited number of patients as long as their general condition permits. Many patients cannot undergo surgery because the metastatic lesion is present at a nonresectable site or multiple metastases are present. [14]. In addition, in patients with liver cirrhosis, thrombocytopenia and elevated INR may be contraindications against surgery. A high model of end-stage liver disease (MELD) score has been shown to be a significant risk factor for severe perioperative complications.

Bodhu et al. [15] have reported that many of the metastasis-promoting genes are embedded in the primary tumors and that the ability to metastasize may be an inherent quality of the tumor from the beginning. The condition of the liver parenchyma and the degree of viral hepatitis mediated liver damage, and the genetic makeup of individuals may have a significant role in the development of metastases. It is also possible that the geographic variation in the gene expression profile of liver parenchyma among HCC patients and/or the differences in the genetic makeup among populations may lead to epidemiologic variations in the metastatic behavior of HCC [8].

Superior sagittal metastasis eroding through the skull masquerading as a clinical lipoma or other soft tissue mass is a rare occurrence. As such, a clinician requires a high degree of clinical suspicious and resultant radiographic evidence in order to make the diagnosis. Ultimately, tissue diagnosis is desirable in such cases, and excision of such lesions should be chosen over percutaneous biopsy due to risk of tumor spread in the biopsy track.

Here, we present the case of a 54-year-old male patient who presented to the surgical clinic with a mobile subcutaneous mass of the vertex of the scalp clinically consistent with a lipoma that turned out to be an erosive metastatic sagittal sinus tumor. We performed a review of the literature with regard to patients with similar HCC lesions.

## 2. Patient and Methods

The case is presented in detail. We conducted a literature review of all the cases between 2009 and 2019 using PubMed using the search words “skull metastases” and “hepatocellular carcinoma”. The following data was extracted: age, sex, clinical presentation, microscopic features, site of metastasis, dural involvement, bone involvement, treatment, outcome, time to expiration (if noted), and presence of viral hepatitis.

## 3. Results

### 3.1. Case Presentation

A 54-year-old male with chronic hepatitis C was referred to our plastic surgery office for a scalp mass. His MELD (model for end-stage liver disease) score was calculated to be 19 just prior to our clinical encounter (bilirubin: 1.5 mg/dl, serum sodium: 134 mEq/l, INR: 1.83, and serum creatinine: 1.3 mg/dl). His vitals were within normal range, and he had no ascites but did have an episode of encephalopathy the previous year which since resolved with improved liver functions. His multiple comorbidities included hypertension, hyperlipidemia, tobacco use, and stage II chronic kidney disease. He had not been diagnosed with any malignancy.

The vertex scalp mass had been gradually increasing in size over the previous month. On inspection, it was noted to be 3 × 4 cm with no visible ulcerations or blisters on the overlying skin. On palpation, the mass was noted to be soft and moderately mobile but with some adherence at the base. The surrounding cranium was palpable except for the area directly under the mass. His cervical lymph nodes were noted to be normal across all subgroups. He had recent head imaging with a computed tomography (CT) scan after a motor vehicle accident 7 months prior. These images were reviewed, and no supracranial nor intracranial mass was noted. Furthermore, there was no sign of any erosive skull pathology.

**Figure 1:**
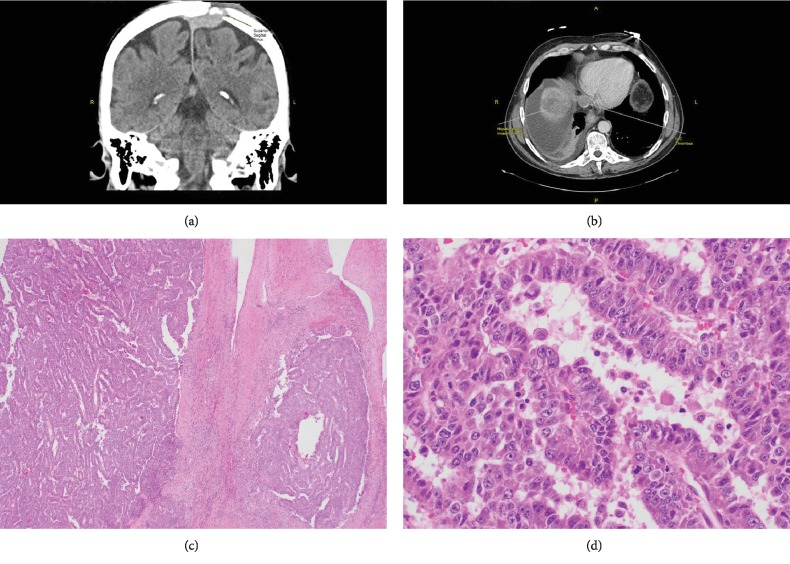
(a) Head CT (coronal view) revealing superior sagittal sinus-based tumor eroding through the cranium. (b) Abdominal CT revealing a heterogenous hepatic lesion invading the inferior venae cava with retrograde extension into the right atrium. (c) Histopathology of cranial tumor showing a characteristic trabecular pattern with thickened cords of cells separated by vascular sinusoids, mimicking the cell plates and sinusoids of a normal liver. (d) Histopathology showing tumor cells with a polygonal shape with prominent nuclei and nucleoli, granular eosinophilic cytoplasm, and intercellular canaliculi resembling liver cells.

As a result, we planned to proceed with excision of the mass, clinically presumed to be a lipoma or cyst, in our clinic procedure room. Under local anesthesia and sterile conditions, an incision was made in the skin over the lesion, and a glistening grey capsule was encountered with a cranially adherent base. There seemed to be a small but present cranial defect. Given the unusual findings, the incision was closed without excision, and a CT and MRI scan of the head was obtained revealing a superior sagittal sinus-based tumor eroding through the cranium (Figure 1(a)). Systemic CT imaging revealed a heterogeneous hepatic lesion invading the inferior venae cava with retrograde extension into the right atrium (Figure 1(b)). With subsequent workup, he was noted to have lung and left 5^th^ rib metastases as well. Neurosurgery was consulted, and an en bloc tumor resection was performed by duroplasty and cranioplasty with bone cement. Microscopically, the tumor revealed a characteristic trabecular pattern with thickened cords of cells separated by vascular sinusoids, mimicking the cell plates and sinusoids of a normal liver (Figure 1(c)). The tumor cells had a polygonal shape with prominent nuclei and nucleoli, granular eosinophilic cytoplasm, and intercellular canaliculi resembling liver cells (Figure 1(d)). The margins were free of tumor and there was no evidence of dural invasion, but there was invasion of tumor into the skull bone. Immunohistochemistry was performed, and alpha-fetoprotein, cam 5.2 and hepar-1, and PAS were positive confirming HCC. The patient was discharged on postoperative day 3 without motor deficits and in good condition. He was due to commence radiation treatment to the rib and skull. The patient unfortunately expired 26 days postoperatively from multiorgan system failure prior to completing this therapy.

### 3.2. Data from the Literature Search

To date, 17 cases of HCC with skull metastases have been reported (Table 1). The mean age of these patients was 57.5 years, ranging from 38 to 81 years. A male predominance (94%) was observed in patients presenting with this metastatic process. Six of the 17 cases had a chronic viral hepatitis prior to skull metastatic presentation which was similar to the diagnosis in our case. We noted that dural involvement with skull metastases was only found in seven previous cases. Only one previous case report demonstrated metastasis to a sinus (cavernous) with our sagittal sinus metastatic presentation only the second in recent literature. 94% of the patients underwent a surgical resection for their skull mass while others were subjected to different treatment modalities including external beam irradiation via intensity-modulated radiotherapy (IMRT), chemoembolization, and radiotherapy. Patient demise was reported in four cases while the remaining patients have had a favorable clinical outcome to the time of publication.

## 4. Discussion

Skull metastasis of HCC is relatively rare, in contrast to the incidence of skull metastasis in the lung, breast, thyroid, and prostate cancers [16]. In the past, the low survival rates of patients with HCC have resulted in low incidences of symptomatic extrahepatic metastases, such as lung and bone metastases (0-5%) [17]. More recent studies have reported that the incidence of bone metastasis from HCC increased to 13% and that the most commonly involved sites were the vertebra, pelvis, rib, and skull [18]. Most HCC patients, however, die of liver failure or internal bleeding without developing clinically apparent extrahepatic metastases [19]. The most common clinical presentation of a skull metastasis from HCC is a painful subcutaneous mass, followed by neurologic deficits, headache, and less commonly seizures. The most common radiologic finding of a skull bone metastasis is the destructive or the osteolytic-type lesion [20].

In a review of 60 articles published between January 1966 and November 2005, Hsieh et al. [21] found the total number of reported cases of HCC with skull metastasis to be 68. The most common presentation is a subcutaneous mass with an occasional painful sensation, which has been reported in 59% of the patients. Cranial nerve deficits, such as visual disturbance dysphagia, deafness, and facial numbness, were present in cases that had skull base involvement.

There are previous reports that document the high frequency of HCC metastases to the head and neck region as an initial presentation of the disease [22, 23] Although it has been theorized that metastases to the head/neck region occurs via the lung [24], there are many cases where metastases in these regions occur without lung metastases on diagnostic imaging [8]. It has alternatively been suggested that metastases could occur without lung metastases either by the vertebral and azygos vein system or by lymphatic systems. Spread via the former route is the preferable one in the case of liver cirrhosis [25]. There have been suggestions that patients who present with a liver cirrhosis history have a higher predisposition for metastases to spread by vertebral vein systems in a retrograde fashion [8].

## 5. Conclusion

HCC with distant metastases markedly worsens the patient's prognosis. It is rarely associated with a metastatic spread to the sagittal sinus eroding to the skull. Diagnosing such a rare clinical entity is a challenging task and is made possible via a high clinical suspicion and resultant radiographic evidence. In patients with a history of liver cirrhosis, an index of suspicious must be maintained regarding the risk of head and neck tumors being the first presentation of metastatic HCC with the probable mode of spread being retrograde via vertebral or azygos veins. This case and our literature review illustrate that skull metastases should be considered in the clinical course of HCC because early diagnosis leads to prompt treatment which can improve patient survival.

## Figures and Tables

**Table 1:** Summary of our PubMed search of all reported cases using search terms “hepatocellular carcinoma” and “skull metastasis” between 2009 and 2019.

Publication	Age	Sex	Clinical presentation	Microscopic features	Site of METS	Dural involvement	Bone involvement	Treatment	Outcome	Time to expiration	Viral hepatitis
Chye et al. [26]	69	F	Acute onset headache	HCC	Left temporal skull	Yes	Yes	Surgery	Uneventful	—	Yes
Hsieh et al. [21]	46	M	Left frontal scalp mass	HCC	Left frontal mass	No	Yes	Surgery	Uneventful	—	No
Woo et al. [27]	46	M	Acute onset headache	HCC	Right temporal	No	Yes	Surgery	Uneventful	—	No
Kim et al. [28]	53	M	Acute onset coma	HCC	Middle fossa floor	Yes	Yes	Surgery	Expired	5 days postop	No
Uei et al. [14]	64	M	Neck pain	HCC	Occipital skull and c-spine	No	Yes	Surgery Radiation	Expired	13 months postop	No
Oshita et al. [29]	81	M	Gait disturbance	HCC	Parietal skull	No	Yes	Surgery	Expired	45 days postop	No
Han et al. [30]	66	M	Acute onset headache	HCC	Left occipital skull	No	Yes	Surgery Chemoembolization	Uneventful	—	No
Ferraz et al. [31]	53	M	Right occipital scalp mass	HCC	Right occipital skull	Yes	Yes	Surgery	Uneventful	—	No
Kim et al. [32]	41	M	Drowsiness Right hemiparesis	HCC	Parietal-occipital skull	No	Yes	Surgery	Uneventful	—	No
Carey et al. [33]	38	M	Headache Right facial paralysis	HCC	Right cavernous sinus	No	Yes	Radiation Chemotherapy	Expiration	7 months posttreatment	Yes
Guo et al. [34]	49	M	Parieto-occipital scalp mass	HCC	Parieto-occipital skull	No	Yes	Surgery Chemotherapy	Uneventful	—	Yes
Tamura et al. [35]	67	M	Pituitary symptoms	HCC	Sella turcica	No	Yes	Surgery Chemotherapy	Uneventful	—	No
Tamura et al. [35]	58	M	Pituitary symptoms	HCC	Sella turcica	No	Yes	Surgery Chemotherapy	Uneventful	—	Yes
Azarpira et al. [36]	38	M	Temporal scalp mass	HCC	Temporal skull	Yes	Yes	Surgery	Uneventful	—	Yes
Brunetti et al. [37]	79	M	Parietal scalp mass	HCC	Parietal-occipital skull	Yes	Yes	Surgery	Uneventful	—	No
Ermis et al. [38]	72	M	Temporal scalp mass	HCC	Parietal-occipital skull	Yes	Yes	Surgery	Uneventful	—	Yes
Fukushima et al. [39]	58	M	Left temporal scalp mass	HCC	Left temporal skull	No	Yes	Surgery	Uneventful	—	No
